# Diabetes-Related Behavior Change Knowledge Transfer to Primary Care Practitioners and Patients: Implementation and Evaluation of a Digital Health Platform

**DOI:** 10.2196/medinform.9629

**Published:** 2018-04-18

**Authors:** Samina Abidi, Michael Vallis, Helena Piccinini-Vallis, Syed Ali Imran, Syed Sibte Raza Abidi

**Affiliations:** ^1^ Medical Informatics Program Department of Community Health and Epidemiology, Faculty of Medicine Dalhousie University Halifax, NS Canada; ^2^ Department of Family Medicine Faculty of Medicine Dalhousie University Halifax, NS Canada; ^3^ Division of Endocrinology and Metabolism Faculty of Medicine Dalhousie University Halifax, NS Canada; ^4^ Knowledge Intensive Computing for Healthcare Enterprises Research Group Faculty of Computer Science Dalhousie University Halifax, NS Canada

**Keywords:** type 2 diabetes mellitus, self-management, health behavior, knowledge management, clinical decision support system

## Abstract

**Background:**

Behavioral science is now being integrated into diabetes self-management interventions. However, the challenge that presents itself is how to translate these knowledge resources during care so that primary care practitioners can use them to offer evidence-informed behavior change support and diabetes management recommendations to patients with diabetes.

**Objective:**

The aim of this study was to develop and evaluate a computerized decision support platform called “Diabetes Web-Centric Information and Support Environment” (DWISE) that assists primary care practitioners in applying standardized behavior change strategies and clinical practice guidelines–based recommendations to an individual patient and empower the patient with the skills and knowledge required to self-manage their diabetes through planned, personalized, and pervasive behavior change strategies.

**Methods:**

A health care knowledge management approach is used to implement DWISE so that it features the following functionalities: (1) assessment of primary care practitioners’ readiness to administer validated behavior change interventions to patients with diabetes; (2) educational support for primary care practitioners to help them offer behavior change interventions to patients; (3) access to evidence-based material, such as the Canadian Diabetes Association’s (CDA) clinical practice guidelines, to primary care practitioners; (4) development of personalized patient self-management programs to help patients with diabetes achieve healthy behaviors to meet CDA targets for managing type 2 diabetes; (5) educational support for patients to help them achieve behavior change; and (6) monitoring of the patients’ progress to assess their adherence to the behavior change program and motivating them to ensure compliance with their program. DWISE offers these functionalities through an interactive Web-based interface to primary care practitioners, whereas the patient’s self-management program and associated behavior interventions are delivered through a mobile patient diary via mobile phones and tablets. DWISE has been tested for its usability, functionality, usefulness, and acceptance through a series of qualitative studies.

**Results:**

For the primary care practitioner tool, most usability problems were associated with the navigation of the tool and the presentation, formatting, understandability, and suitability of the content. For the patient tool, most issues were related to the tool’s screen layout, design features, understandability of the content, clarity of the labels used, and navigation across the tool. Facilitators and barriers to DWISE use in a shared decision-making environment have also been identified.

**Conclusions:**

This work has provided a unique electronic health solution to translate complex health care knowledge in terms of easy-to-use, evidence-informed, point-of-care decision aids for primary care practitioners. Patients’ feedback is now being used to make necessary modification to DWISE.

## Introduction

### Background

An estimated 9 million Canadians are living with diabetes, prediabetes, or undiagnosed diabetes [1]. Effective diabetes self-management, which relies on the behavior of an individual [2,3], shows great potential, as it can improve outcomes, decrease the risk of complications, and reduce diabetes-related hospitalizations and costs. Optimal diabetes control requires ongoing adherence to medication and diabetes care, self-monitoring of blood glucose, achieving a healthy weight, eating healthy, abstinence from smoking, moderate alcohol consumption, being physically active, and managing stress. The goal of diabetes self-management intervention is to support the individual to achieve positive behavior changes for achieving optimal diabetes control. Behavioral science is now being integrated into diabetes self-management interventions [2-4] to better educate and engage individuals in the self-management of their condition. In this regard, theory-driven, evidence-based behavior change approaches have been applied to (1) increase motivation to change when it is low, using the stages of change model [5,6], theory of planned behavior [7], social cognitive theory [8,9], and motivational interviewing techniques[10]; (2) support effective behavior change when motivation is present [11]; and (3) address emotional and relational barriers to behavior change [12,13]. Behavior change interventions have been developed and applied to chronic disease management [14-17], and more specifically to achieve diabetes control [18-20]. Diabetes management requires an interprofessional team effort, with the family physician as an initial and long-term health care provider, diabetes specialists providing therapeutic support, diabetes educators providing assistance to achieve diabetes control, behavior change experts influencing positive self-management behavior, and finally, with the patient as the most integral member of this team. Canadian Diabetes Association’s (CDA) Clinical Practice Guideline (CPG) [21] also suggests an interdisciplinary team approach toward diabetes management. Generally, diabetes in Canada is managed by PCPs, with auxiliary support provided by nurses and dietitians qualified by the CDA as certified diabetes educators (CDEs) in diabetes management centers (DMCs). Patients are referred to a DMC by their PCP. At a DMC, patients are provided with self-management education and tools to help them self-manage their condition and associated risk factors. The CDEs work closely with PCPs.

Although PCPs are heavily involved in the long-term care of their patients with diabetes, studies have shown suboptimal and nonstandardized diabetes care at the primary care level [22-24]. Despite the availability of specialized behavior change interventions and evidence-based CPG on diabetes management, the challenge is how to translate these knowledge resources during care so that PCPs can easily use them to offer evidence-informed behavior change support and diabetes management recommendations to patients with diabetes. The Behavior Change Institute (BCI) [25] at Nova Scotia Health Authority in Halifax offers PCPs and CDEs with behavior change training and support to help them educate patients who require assistance in modifying unhealthy behaviors and need guidance to self-manage their chronic condition. However, it is also noted that because of resource constraints, there are limited competency-based behavioral support training opportunities available for PCPs [26-28], and likewise, there are limited opportunities for patients to access the services provided by DMCs [29]. Given the challenges faced by both PCPs and patients to access behavior change programs, we argue that it is prudent to leverage digital health technologies to (1) deliver to PCPs CDA’s CPG-based diabetes care decision support as well as behavior change interventions planning support to help them manage both the clinical and behavioral aspects of diabetes control and (2) empower patients to better self-manage their diabetes through personalized behavior change interventions accessible to them by mobile technologies. To achieve these functional objectives, working in collaboration with BCI, we have developed computerized behavior change training modules for PCPs as well as patients with diabetes to improve diabetes control outcomes.

### Objectives

In this paper, we present an innovative computerized decision support platform to (1) assist PCPs in administering evidence-based behavior change strategies and CPG-based recommendations for diabetes management and (2) empower patients with the skills and knowledge required to self-manage and monitor their diabetes through planned, personalized, and pervasive behavior change strategies. The key research tasks pursued in this project include (1) the development of a behavior change strategy based on evidence-based theories to better engage, empower, and inform the PCPs and their patients about behavior change strategies pertaining to diabetes control; (2) formulation of a comprehensive and validated knowledge base (in terms of a high-level behavior change ontology) that encapsulates semantic associations between multiple elements, that is, patient profile, CPG-derived diabetes management recommendations, and behavioral theory constructs that are coupled with behavior change strategies; (3) implementation of an integrated clinical decision support and behavior change intervention planning framework called Diabetes Web-centric Information and Support Environment (DWISE) that leverages semantic Web technologies to computerize behavioral and clinical knowledge in terms of an ontological knowledge model and generate personalized behavior change strategies by reasoning over the computerized knowledge using the patient profile. DWISE can be accessed by PCPs via a secure Web interface, and patients can access it via the DWISE mobile app; (4) evaluation of DWISE in terms of its usefulness, usability, and functionality through qualitative studies involving PCPs and patients. In the subsequent sections, we discuss in detail the design, development, and evaluation of DWISE.

### Problem Description and Solution Rationale

CDA’s CPG [21] recommends that individuals with diabetes manage their disease with the help of an integrated diabetes health team while using a self-management model that incorporates knowledge and skills development coupled with cognitive behavioral interventions. Furthermore, it is recommended that individuals (and their families) with diabetes should be regularly screened for symptoms of psychological distress, and preventive interventions such as participative decision making, feedback, and psychological support should be incorporated within diabetes self-management interventions. Although DMCs exist across Canada, approximately 70% of individuals living with diabetes are unable to benefit from a DMC because of access limitations; rather, they may receive care from their PCP [22]. Several Canadian [22-24] and international studies [30] have found suboptimal management of type 2 diabetes in primary care settings, including suboptimal glycemic control [23,24] and failure to achieve CPG-recommended targets for glycated hemoglobin (HbA_1c_) and low-density lipoprotein cholesterol in patients with type 2 diabetes [22,24,30]. On observation, the recommendations proposed by the CDA are currently not being implemented in the diabetes care process because of (1) lack of access to psychosocial resources within diabetes medical services where PCPs are not well equipped to manage behavior change in individuals with low motivation or who face psychosocial barriers to change and (2) lack of access to diabetes management CPG during care. Given the limited training opportunities to empower PCPs to administer behavior change interventions [26-28] and also limited patient access to the DMC and behavior change support [29], we argue that new knowledge transfer approaches need to be implemented to overcome this prevailing knowledge gap, and digital health technologies should be leveraged to administer behavior change programs that can potentially help to improve diabetes control outcomes.

**Figure 1:**
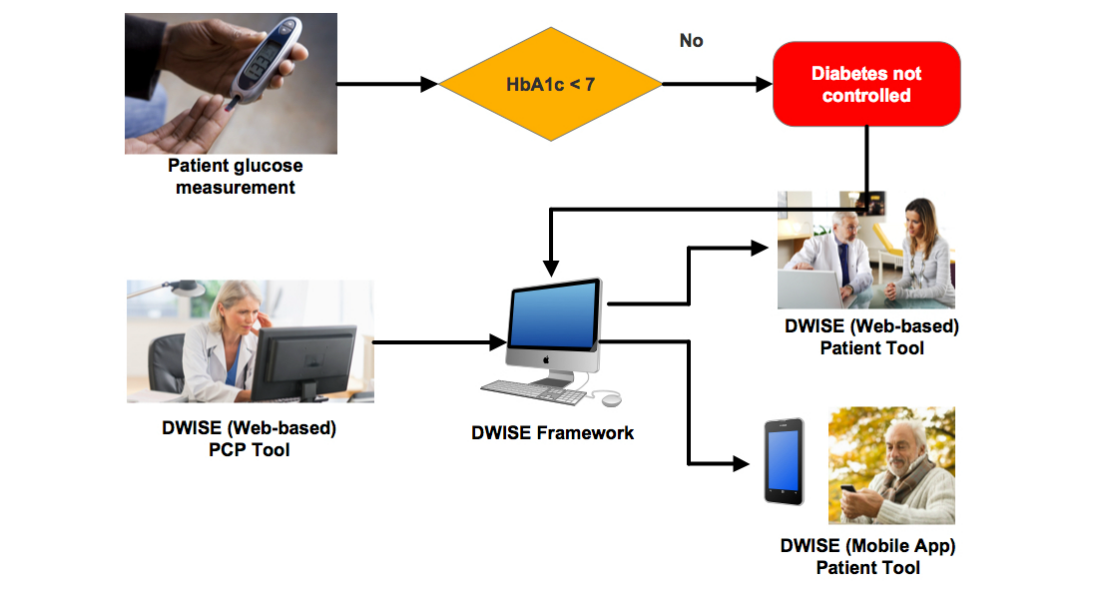
Diabetes Web-Centric Information and Support Environment (DWISE) framework overview. HbA_1c_: glycated hemoglobin.

To address the gaps in diabetes-related behavior change knowledge transfer, in this project, we demonstrate the applicability of digital health technologies to (1) provide decision support for PCPs to design and administer personalized behavior change strategies and (2) simultaneously provide motivational and educational support for patients with diabetes to self-manage their condition for improving diabetes control outcomes. In this regard, we present a knowledge management–based approach, together with its implementation and deployment, in terms of a DWISE that features the following functionalities: (1) assessment of PCPs’ readiness to administer validated behavior change interventions to patients with diabetes; (2) educational support for PCPs to help them offer behavior change interventions to patients with diabetes; (3) access to evidence-based material, such as the CDA’s CPG, to the PCPs; (4) development of personalized patient self-management programs to help patients with diabetes achieve healthy behaviors to meet CDA targets for managing type 2 diabetes; (5) educational support for patients to help them achieve behavior change; (6) monitoring the patients’ progress in adhering to their behavior change program and motivating them to be in compliance with their program. DWISE offers these functionalities to PCPs through an interactive Web-based interface, whereas the patient’s self-management program and associated behavior interventions are delivered through a mobile patient diary via mobile phones and tablets (Figure 1). A key feature of our solution is the incorporation of semantic Web-based knowledge modeling and execution technologies [31,32] that are applied to translate diabetes CPG and behavior change knowledge resources in terms of point-of-care decision support and mobile self-management support resources for PCPs and patients, respectively.

### Diabetes Web-Centric Information and Support Environment Solution Approach

We contend that health education and support for chronic disease self-management should not just focus on changing the patient’s awareness of the disease but should also help to empower the patient to make the right choices to achieve effective disease management via self-management support mechanisms. In this regard, our solution approach is to incorporate validated behavior change theories—in our case social cognition theory (SCT) [33]—to address an individual’s self-efficacy expectations and perceived capabilities to perform self-care actions. Self-efficacy attainment has been shown to influence an individual’s motivation, accomplishments, self-regulation, and efforts to perform self-care actions [33]. Patient education programs grounded in self-efficacy theory have been shown to enhance patient’s adherence to self-care behavior, which in turn has been shown to improve clinical outcomes [34-37]. On the basis of the principles of SCT, our approach is to develop a specialized behavior change strategy that first assesses the PCP and patient’s readiness to undertake behavior change interventions, and then, in response to their readiness levels, stipulate a personalized behavior change program. Although there are several existing self-management programs that target patients’ behaviors, a unique aspect of our solution approach is the assessment of PCPs’ readiness and in turn enhancement of their self-efficacy to administer behavior change counseling to patients who are facing psychosocial barriers to change behaviors that are affecting their condition. We argue that to implement an effective behavior change program at the primary care level, it is important to initially assess PCPs’ readiness and self-efficacy to administer behavior change counseling and then provide them necessary training and decision support services so that they can effectively administer personalized behavior change counseling to their patients. Therefore, a unique aspect of our digital health solution approach is the provision of PCP-focused behavior change readiness assessment and decision support services that help PCPs to achieve self-efficacy in behavior change counseling.

The DWISE solution has 2 main elements: (1) behavior change strategies that are grounded in theoretical behavior change models [38,39] and (2) knowledge translation or transfer methods that are guided by a pragmatic health care knowledge management approach [40].

Our solution involves modeling and computerization of diabetes management–related clinical and behavior change knowledge sources using semantic Web methods and then using digital health technologies to design personalized behavior change interventions and deliver behavior change support using Web and mobile interfaces [41-44]. For knowledge modeling and computerization, we have pursued an ontology-based knowledge modeling approach [45] to develop a behavior change ontology (BCO) [41] that semantically represents and integrates the BCI’s behavior change strategies, CDA’s diabetes management CPG recommendations, and the SCT behavior change model; in this way, the BCO both computerizes and translates these knowledge sources in terms of actionable behavior change programs. We used the Web Ontology Language (OWL) [46], a computational logic-based language to develop the BCO. Our ontology-based behavior change modeling approach is novel and, in practice, we achieved an integrated knowledge model that entails (1) sections of the CDA’s CPG pertaining to the management of glycemic control and (2) elements of BCI’s behavior change strategy, including readiness to change assessments, motivational enhancement interventions, and self-efficacy attainment and self-management. The BCO is used to personalize behavior change strategies at 2 broad levels: (1) clinical level, where CPG-derived clinical variables are used to tailor most relevant recommendations for the given patient and (2) behavioral level, where the behavioral variables derived from the relevant behavioral models are used to personalize behavior change interventions.

## Methods

### Overview

To develop DWISE and, in particular, its knowledge backbone (ie, the BCO), we used an ontology-based information system design framework called Methontology [47] that stipulates a cyclical ontology engineering approach, which involves experts to validate each iteration of the knowledge model. We discuss the different aspects of DWISE design and development in the following paragraphs.

### Behavior Change Knowledge Modeling

The purpose of the behavior change knowledge modeling step is to develop specialized behavior change algorithms targeting the specific needs of both the PCPs and patients, which will be used to formulate theory-driven behavior change strategies. To develop behavior change algorithms based on our knowledge management approach, we abstracted and modeled key behavior change knowledge constructs from the available knowledge sources—the BCI strategies, diabetes CPG, and SCT model.

**Figure 2:**
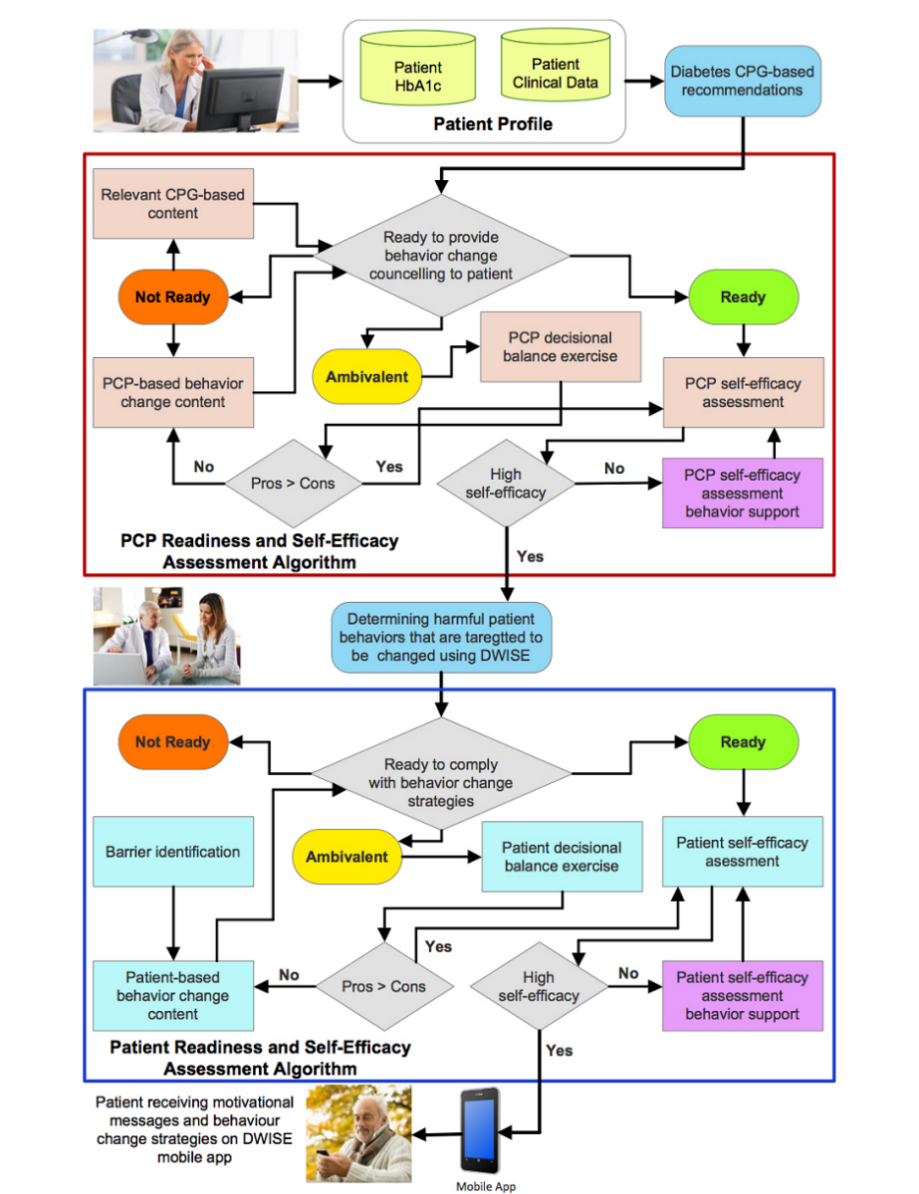
High-level PCP and patient behavior change algorithm. CPG: clinical practice guidelines; ; HbA_1c_: glycated hemoglobin; DWISE: Diabetes Web-Centric Information and Support Environment; PCP: primary care practitioner.

Knowledge modeling involved the abstraction of clinical and behavioral determinants from the paper-based knowledge sources that influence diabetes control outcomes. These determinants were then systematically linked and represented in terms of a rich multidimensional patient behavior profile. To enhance the ability of the patient’s behavior profile to personalize a behavior change strategy, the behavioral determinants were represented using multiple levels or ranges to capture the nonlinear nature of behavior change among different individuals. We developed 2 high-level behavior change algorithms, one each for PCPs and patients (as shown in Figure 2).

Each algorithm systematizes a variety of assessment tools based on behavioral determinants, range of PCP and patients’ inputs (in terms of observations, goals, and preferences), behavior change strategy options, behavior change strategy elements, motivational messages, and education material. The algorithms are based on 3 behavior change models that are described in the following paragraphs.

A behavior change readiness assessment model was developed by our team at the BCI to assess the readiness levels of both PCPs and patients toward behavior change programs. When used in the PCP’s behavior change strategy tool, our model assesses the readiness of a PCP to provide behavior counseling to help modify harmful behaviors in patients. When used in the patient’s behavior change support tool, the model measures the readiness of a patient to comply with recommended self-management support strategies. The behavior change readiness assessment model uses a systematic questionnaire (with responses of “yes,” “no,” and “maybe”) to categorize an individual into 3 stages of readiness, that is, ready, ambivalent, and not ready. As noted in other stage models, for instance, the transtheoretical model [48], the transition between readiness stages is nonlinear and is dependent on the levels of motivation and self-efficacy that can be improved by cognitive and behavioral therapies. To account for the nonlinear progression in behavior change, we included 2 additional behavior models—a decisional balance measure and a self-efficacy assessment model—to our behavior change readiness assessment model.

We used the decisional balance measure to determine an individual’s perception about the expected benefits (pros) of modifying a behavior as opposed to the disadvantages or costs (cons) of this behavior change. An individual who is deemed ambivalent or not ready for behavior change programs is required to undergo a decisional balance assessment, which includes up to 5 pros and 5 cons that measure positive and negative perceptions of PCPs in administering self-management behavior change support, and that of patients in adopting self-management behaviors. Decisional balance assessment is repeated after educating the ambivalent or not ready individual about the benefits of behavior change programs.

After the decisional balance assessment where the individual has been assessed to be ready for behavior change programs, a self-efficacy assessment is performed to measure a “ready” PCP’s degree of confidence that he or she can administer behavior change counseling, as well as a “ready” patient’s degree of confidence in complying with behavioral change strategies.

In practice, the PCP behavior change algorithm initially performs behavior change readiness assessment, decisional balance assessment, and self-efficacy assessment, and in response to the assessment, a range of targeted educational material is provided to the PCP. During a patient encounter, the PCP behavior change algorithm supports the design of a personalized behavior change strategy by providing targeted CPG-based recommendations, most suitable behavior to be targeted for a patient, and the corresponding behavior change strategy that is tailored based on the Specific, Measurable, Action-Oriented, Relevant, Timely (SMART) goals jointly set by the PCP and the patient and the patient profile.

The patient’s behavior change algorithm generates the patient support material based on the SMART behavior change goals to help the patient achieve the recommended HbA_1c_ level. The patient behavior change algorithm operationalizes the personalized behavior change strategy to generate and deliver motivational messages, educational material, and recommendations for overcoming barriers to change so that patients can achieve their SMART goals.

### Ontology-Based Modeling of Behavior Change Knowledge

**Figure 3:**
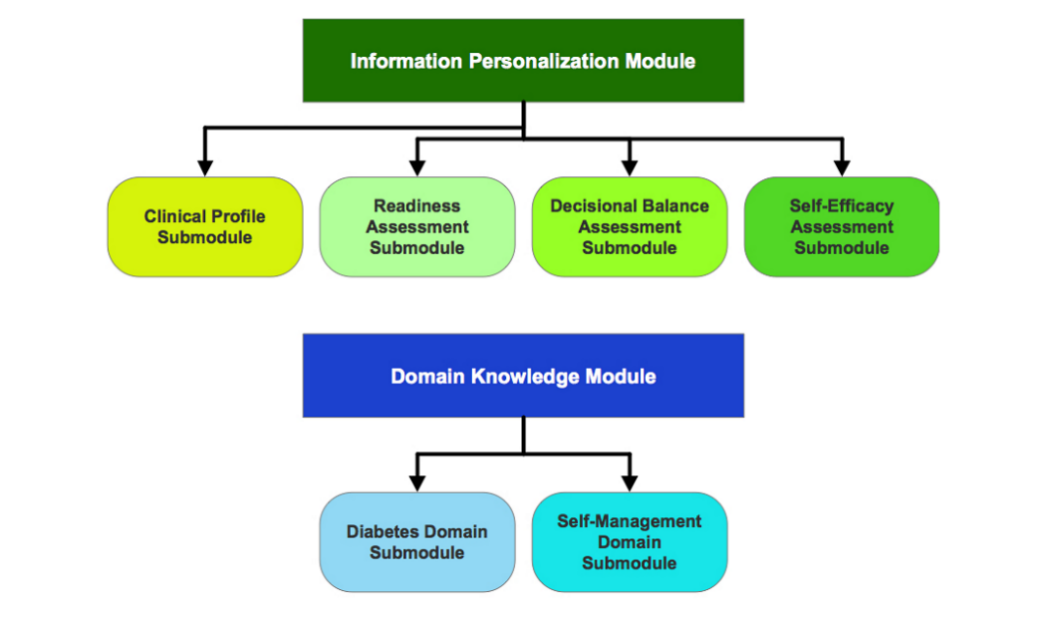
Information personalization and domain knowledge elements of Behavior Change Ontology.

To formally represent the behavior change knowledge model (and the algorithms), we developed a high-level BCO [41] using ontology modularization principles [49,50] (see Figure 3), resulting in distinct yet interconnected knowledge modules representing the different aspects of behavior change programs. Ontology modularization approach reduces the complexity of developing a large multifaceted ontology, and we could handle the complexity of formally representing and integrating multiple behavior change models, assessment tools, behavior change strategies, and the CPG for diabetes management in a single, comprehensive behavior change knowledge model. We used the Methontology [47] methodology to develop the 2 distinct yet integrated knowledge modules within BCO, which are as follows (Figure 3):

- Information Personalization module that is used to create patient behavior change profiles, and it consists of 4 knowledge submodules:
  - Clinical Profile module represents clinical attributes derived from the CDA’s CPG, pertinent to describing the patient’s clinical medical profile with respect to diabetes control.
  - Readiness Assessment module represents the behavior change readiness assessment strategy for both PCPs and the patients, as developed by our team at BCI.
  - Decisional Balance Assessment module represents the positive and negative perceptions of “not ready” and “ambivalent” PCPs.
  - Self-Efficacy Assessment module represents the SCT-based self-efficacy assessments of PCPs and patients.

The patient behavior change profile is dynamically created when the information personalization module is executed using the patient’s attributes.

- Domain Knowledge module that is used to represent the domain (ie, behavior change and self-management for diabetes control) knowledge, and it comprises 2 submodules:
  - Diabetes Domain module represents the evidence-based diabetes control recommendations as stipulated by CDA’s CPG.
  - Self-Management Domain module represents SCT-based self-management knowledge, for example, barriers to diabetes self-management and behavior change, self-management and behavior change support materials and strategies, and SMART goal setting support.

**Figure 4:**
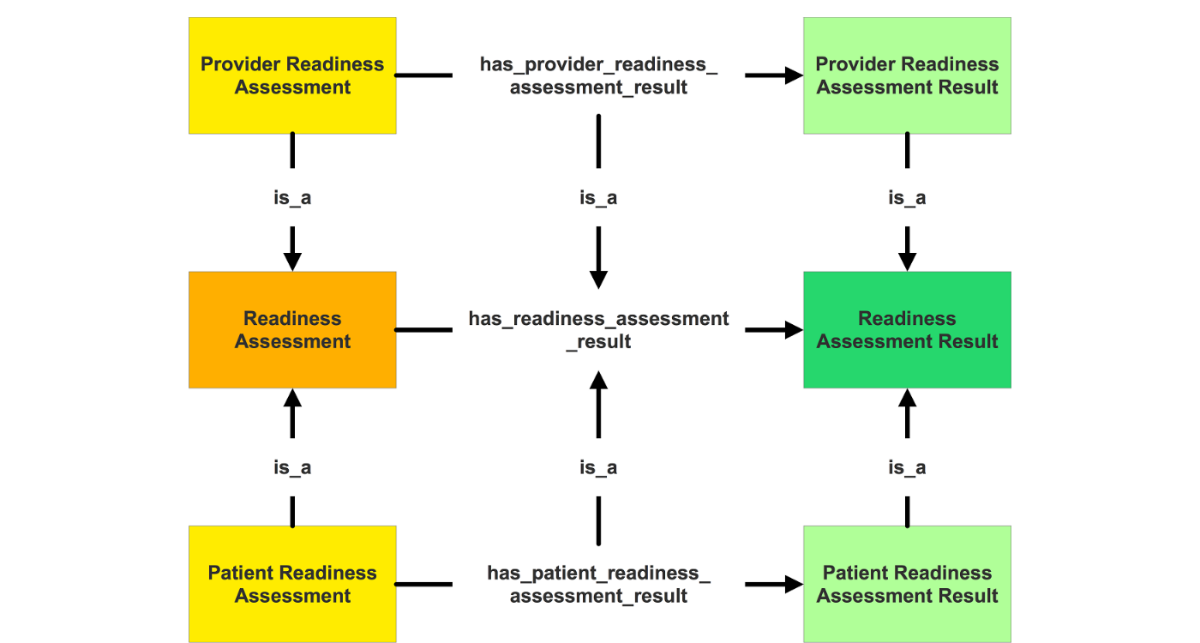
A subset of readiness assessment module in Behavior Change Ontology, depicting procedural relationships between classes “Readiness Assessment” and “Readiness Assessment Result.”.

To develop the BCO, we used object properties to represent declarative and procedural relationships between classes. Axioms and rules were used to augment the procedural aspect of the BCO further. Figure 4 shows a subset of Readiness Assessment module in the ontology, depicting procedural relationships between the classes “Readiness Assessment” and “Readiness Assessment Result.”

Information personalization submodules—readiness assessment, decisional balance assessment, and self-efficacy assessment—computerize assessment questionnaires to identify the patient’s current behavioral predisposition. The questionnaires are represented as an object and the questions within them as their properties. The questions’ responses such as values from 1 to 4 are represented as datatype properties, whereas questions that require a predefined statement as a response (eg, what is the highest level of education you have achieved?) are represented as object type properties. Property restrictions, such as cardinality restrictions, range (both for object type and data type), and allowed values were used to ensure knowledge integrity. The Information Personalization module contains 10 classes (Barrier, Barrier to Change, Clinical Profile, Decisional Balance, Decisional Balance Result, Readiness Assessment, Readiness Assessment Result, Self-Efficacy Questionnaire, Self-Efficacy Result, and Prognostic Factors). In total, BCO represents 18 top-level classes, whereas the entire class hierarchy consists of 80 classes. There are 16 top-level object properties with 18 object subproperties and 7 top-level data type properties with 40 data type subproperties. Finally, BCO was instantiated using the content that was gathered and developed in the PCP and patient algorithms. We used Protégé 2000 knowledge acquisition tool (Stanford Center of Biomedical Informatics Research, Stanford University, Stanford, California) [51] as the ontology editor using the OWL [46].

BCO was evaluated for (1) knowledge accuracy and utility by 3 domain experts (psychologist, endocrinologist, and a family physician) and (2) semantic accuracy to ensure logical consistency. Although experts generally agreed with the representations, they nevertheless suggested a few improvements, for example, better definition of the class Clinical_Profile in terms of its properties and relationship with the class Clinical_Parameter. Changes were made to BCO in terms of its concept description, relationships, and constraints after each evaluation event in response to the experts’ comments. The technical evaluation of BCO was carried out in accordance with the criteria suggested by Gomez Perez [52], which include the 3 Cs: Consistency, Completeness, and Conciseness. FaCT++ [53], an open-source OWL DL reasoner, was used to perform the subsumption tests on BCO to establish its concept satisfiability and consistency. Fact++ was also used to compute the inferred class hierarchy and identify redundant arcs between the classes. Our classification tests did not indicate any redundant arcs in BCO; therefore, it is concluded that the asserted hierarchy is similar to the inferred hierarchy. Finally, in terms of the evaluation of the necessary and sufficient conditions of a predicate, domain and range of relations, and generalization and specialization of classes, it was noted that BCO demonstrated representational capacity to adequately instantiate all the domain concepts and relationships captured in the behavior change algorithms and CPG. The complete BCO can be accessed at the Github website [54].

### Implementation of Diabetes Web-Centric Information and Support Environment

**Figure 5:**
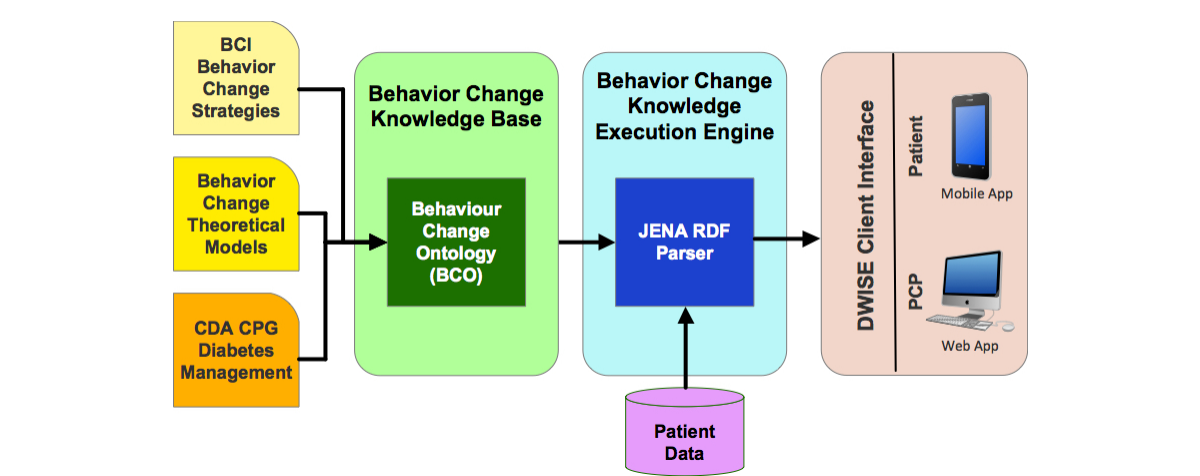
Diabetes Web-Centric Information and Support Environment (DWISE) technical architecture. BCI: Behavior Change Institute; CDA: Canadian Diabetes Association; CPG: clinical practice guidelines; PCP: primary care practitioner.

A prototype of DWISE framework consisting of the Web-based PCP interface and a mobile app for patients has been implemented. The overall technical architecture of DWISE framework is illustrated in Figure 5. The Web application is written in Java and Vaadin, and information is stored in a relational database on a secure centralized server. Within DWISE, BCO serves as the main knowledge resource—an open-source Java library for OWL, and RDF is used to read and manipulate the domain knowledge contained in BCO’s OWL files. The main BCO elements—resources, properties, and property values—are used to model the temporal relations inherent in DWISE’s information flow. The decision rules are translated into JENA rule syntax, which are input into the JENA reasoning system; JENA uses the rules and temporal relations specified in the BCO to integrate ontological modules during execution of the BCO to infer knowledge-based decision support and behavior change strategy planning based on patient profile. We have leveraged our existing work in semantic Web technologies for knowledge representation [55-60], and we exploit our OWL-based reasoning methods [61] to infer a dynamically generated patient-specific behavior change strategy.

#### Diabetes Web-Centric Information and Support Environment Web-Based Decision Support System for Primary Care Practitioners

**Figure 6:**
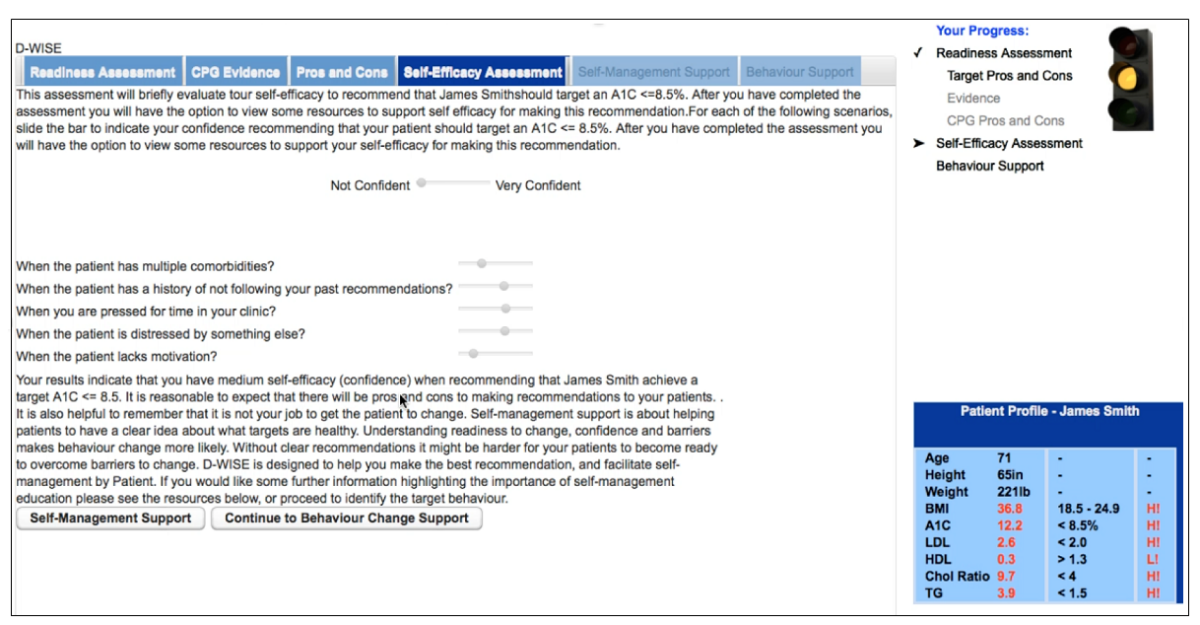
Tool for primary care practitioners assessing self-efficacy of the family physicians/certified diabetes educators.

DWISE decision support framework consists of a PCP and a patient tool. DWISE PCP decision support tool (Figure 6) provides recommendations from the CDA’S CPG (eg, most appropriate target HbA_1c_ for a patient) that are tailored toward the clinical profile of the patient for whom behavior change support is being sought. In addition, the PCP decision support tool also assesses the readiness and self-efficacy of the PCP to administer behavior change counseling to help patients achieve the CPG-stipulated diabetes control targets by changing harmful behaviors. For PCPs who are deemed as “ready” to administer behavior change counseling, DWISE enables the PCP to engage their patients in a shared decision-making setting to develop a personalized behavior change strategy, akin to behavior change consultations performed at the BCI. The strategy is tailored toward a patient’s behavioral profile that is formulated through a series of behavioral assessment exercises administered through DWISE in a shared decision-making setting.

#### Diabetes Web-Centric Information and Support Environment Patient Support Mobile App

**Figure 7:**
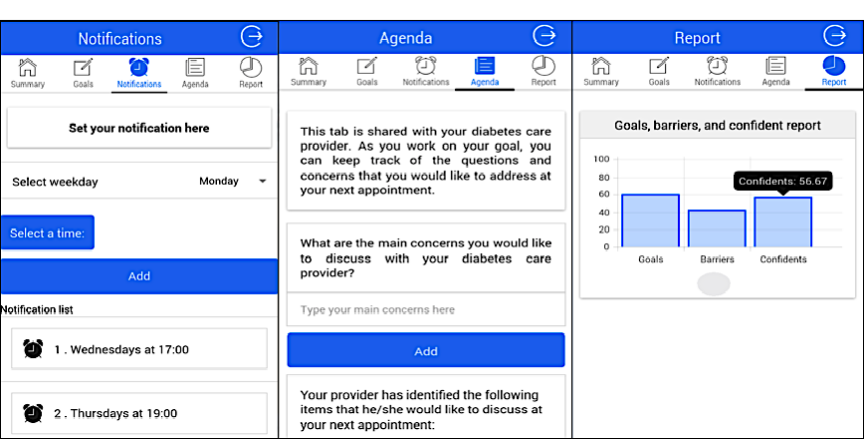
Screenshots from Diabetes Web-Centric Information and Support Environment (DWISE) mobile apps depicting functionalities such as scheduling, notifications, barrier identification, and feedback.

DWISE patient support tool is implemented as both a Web-based system and a mobile app, with the functionality to deliver the following self-management support to patients: (1) behavior change strategies such as goal setting, behavior shaping, stimulus control, and reinforcement management; (2) context-aware motivational and behavior change educational messages; and (3) communication with care providers. The DWISE mobile app (Figure 7) is designed to support the patient to enact the self-management plan that he or she has formulated with the PCP. The DWISE mobile app features are (1) patient diaries for capturing vitals, diet, exercise, stress, and mood; (2) proactive alerts to underline stimulus control and reinforcement management; (3) context-aware motivational and behavior change educational messages and reminders to help the patient adhere to the self-management schedule; and (4) communication with the care providers. The DWISE mobile app has been developed for the Android platform using necessary data security and privacy regulations, with provisions for a future iOS-based app implementation.

### Evaluation of Diabetes Web-Centric Information and Support Environment

Two qualitative studies that incorporated Think Aloud Protocol (TAP) method [62,63] were conducted to evaluate DWISE’s usability by PCPs and patients. In addition, a focus group study was conducted to examine the perspectives of the PCPs and patients about the usefulness of DWISE for diabetes self-management in shared decision-making environment.

#### Study Design

For the usability study, we used a cognitive and usability engineering framework [62] to establish whether DWISE meets the functional goals, content suitability or comprehensiveness, and usability needs of PCPs and patients. Furthermore, our intent was to identify the usability- and content-related issues that need to be resolved in the next version of DWISE and to receive the end-user feedback.

After ethics approval, we randomly recruited 10 PCPs (4 family physicians and 6 CDEs) for the PCP study and 11 patients for the patient study. The sample size estimate is based on the evidence that 70% of severe usability problems can be uncovered within the first 5 users, and up to 85% by the eighth user [62]. The PCP study included PCPs, family medicine residents, and CDEs licensed in Canada and practicing in Halifax. Adult patients with diabetes who were residing in Halifax were included in the patient study. Exclusion criteria included being on the DWISE research team and lack of proficiency in English (because DWISE is currently developed in the English language). Both groups were asked to complete a demographic and background questionnaire. Each PCP/CDE was provided with 3 standard case scenarios that simulated 3 different patients to interact with DWISE, thus yielding 30 TAPs from 10 PCPs.

**A priori categories for focus group data analysis.**

1. Barriers to Diabetes Web-Centric Information and Support Environment (DWISE) usage
2. Facilitators to DWISE usage
3. Patient behavior change
4. Patient empowerment and education
5. Patient autonomy and preference
6. Patient-provider communication
7. Encounter-related issues
8. Professional roles and responsibilities
9. Usability- or acceptability-related issues

Patients were presented with a standard behavioral recommendation, that is, physical activity that they have hypothetically agreed to pursue in concert with their PCP/CDE. The patients then defined a specific target behavior, assessed their readiness, and received support in setting a goal for behavior change. This yielded 11 TAPs from 11 patients. During the interactions with DWISE, each participant was encouraged to think aloud. Participants’ screen activity and audio were recorded using the QuickTime player. The usability study design is presented in the study by Abidi et al [64] in detail.

For the focus group study, a purposive sampling strategy was used to recruit 4 patients and 3 PCPs, after acquiring the ethics approval. The purpose of this study was to engage patients and PCPs in shared decision-making environment to elicit (1) initial impression about the DWISE system; (2) advantages and disadvantages of DWISE in providing CPG-based recommendation and behavior change strategies to the PCPs and patients; (3) potential impact of DWISE on patient-provider communication and relationship around diabetes-related behavior change; and (4) suggestions to improve DWISE. The session was audio-recorded and transcribed verbatim and was supplemented by field notes, sketches, and observation logs. The experts on the team prepared a semistructured moderator’s guide based on their clinical and research experience and the review of the related literature. The guide included open-ended questions and problem-based representative scenarios related to various self-management processes to stimulate conversations in case of unresponsive participants. Content validity of the guide was established by review of the literature on diabetes self-management in populations that are culturally and socioeconomically similar to the population of interest. Further validity was established through critique, change, and consensus of the expert research team members.

#### Data Analysis

Computer screen activity, audio files transcribed verbatim, field notes, and observation logs were analyzed in the ATLAS.Ti software (ATLAS.ti Scientific Software Development GmbH) using inductive thematic coding [65] method. Ten PCPs with 3 case scenarios yielded 30 TAPs, and 11 patients with 1 case scenario generated 11 TAPs. Segments of interesting audio and screen activity data were selected as quotations, which were professed as unit of analysis. Open codes were directly applied to the quotations. ATLAS.Ti also calculated code frequency, that is, number of quotations to which a particular code is applied. Large numbers of quotations associated with a code indicate strong evidence found for this code, which in turn endorses the “groundness” of that code in the data. Axial coding was then applied to draw categories from the open codes based on commonality between them. To perform thematic analysis on the focus group data, a priori categories (Textbox 1) based on the open-ended questions in the moderator’s guide were established. During data analysis, the open codes assigned to the quotations were classified as axial codes based on their commonality. The axial codes were constantly compared against a priori categories listed in Textbox 1 and assigned to one or more of these categories. Validation of the identified open and axial codes was performed by continual referral back to the original computer screen activity, audio files, transcripts, and observational notes. Furthermore, another researcher on the team reviewed the data, so that any conflicts or discrepancies were resolved through discussion and consensus before the codes were finalized.

## Results

### Participants’ Demographics and Background

The background and demographic information of PCPs and patients, which was collected for the TAP study, is presented in detail in the study by Abidi et al [64]. In general, both the PCP and patient samples were biased toward women and those comfortable with computers. The spread of experience for PCPs was uniform. Although all of them used electronic medical records, they were not particularly experienced with respect to the use of decision support systems. Their comfort level with administering behavior change strategies in terms of experience and confidence was found to be variable. Median age of patients was 52 years. The patient sample was slightly more educated than the general population and was predominantly urban. All patients had previous behavior change experience and generally were somewhat confident in using behavior change strategies.

The 7 participants for the focus group study included 3 CDEs and 4 patients. All 3 CDEs were females, and of 4 patients, 3 were females, and 1 was male. Ages for CDEs ranged from 29 to 55 years, and patients were aged between 49 and 64 years. All CDEs worked at DMCs. CDEs had a median of 11 years of experience (range 3-19 years). Patients had diabetes for a median of 13.5 years (range 2-25 years).

### Qualitative Results From Primary Practitioner Tool Study

**Figure 8:**
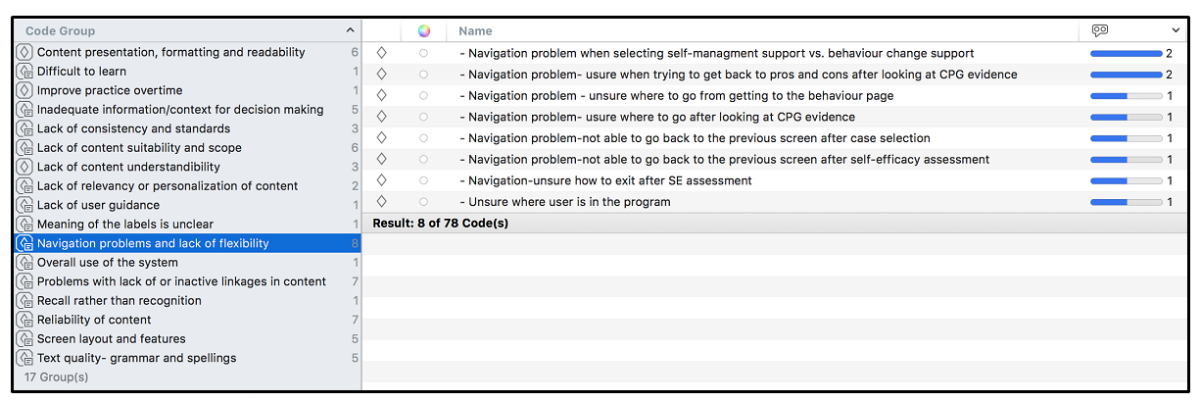
Axial codes in a primary care practitioner;'s Think Aloud Protocol.

In total, 31 independent open codes based on usability issues were identified in TAPs by 30 PCPs. The detailed results are presented in the study by Abidi et al [64]. Several of these codes occurred more frequently in the data, for example, “Need more patient information for pros and cons” occurred in 19 quotations and “Need more information for readiness assessment” occurred in 11 quotations. In total, 17 themes of axial categories of usability issues emerged based on the commonalities noted in the open codes (Figure 8). For example, the axial category “Navigation Problems and Lack of Flexibility” (Figure 8) had 8 codes, and one of the codes in this category, that is, “Navigation Problem-Unsure when trying to get back to pros and cons after looking at CPG evidence” occurred in 2 quotations. Example of one such quotation is “Is readiness assessment the one where I came from...so should I go there.”

### Qualitative Results From Patient Tool Study

**Figure 9:**
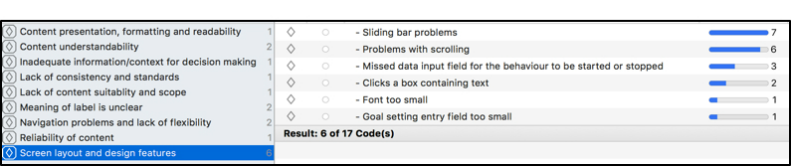
Axial category “Screen layout and design features” contain 6 open codes.

Patients’ TAPs yielded 17 open codes and 9 axial categories of usability issues. The detailed results are presented in study by Abidi et al [64]. Figure 9 shows the axial categories of usability problems with the number of codes in each axial category. Of 17 open codes, most critical codes include “Unsure of goal setting data entry field,” which occurred in 11 quotations; “Sliding bar problems,”which occurred in 7 quotations; and “Problems with scrolling,” which occurred in 6 quotations. An exemplar quotation that contains the code “unsure of goal setting data entry field” is as follows: “I have entered my goal and now it is asking me to be specific...so it should already be somewhere...”

Each axial code represents areas for possible enhancement needed in DWISE content, screen layout and features, design, and other usability features. Sorting by theme and related codes and their frequency gives a detailed picture of the specific improvements and modifications that are needed before DWISE can be tested for its efficacy. The criticality of usability problems is based on the number of open codes in each category and the frequency of these codes in the quotations.

We considered the criticality of a code and the axial category to improve the design of DWISE. Any open code that occurred in a single quotation was discarded. In general, for the PCP tool, most problems were associated with the navigation of the tool and the presentation, formatting, understandability, and suitability of the content in the tool. For the patient support tool, most issues were related to the tool’s screen layout and design features, understandability of the content, clarity of the labels used, and navigation across the tool. We used this feedback from the qualitative study to modify DWISE in terms of its user interface design and its content.

### Salient Findings From the Focus Group Study

In all, 73 open codes were discovered in data that were classified into 27 independent axial codes. Table 1 shows the a priori categories and axial codes assigned to these categories. The number in parenthesis next to each axial code indicates the number of open codes contained in each axial code, thereby indicating groundness of each axial code in the data. These categories are explained in an integrated manner, that is, from both the patients’ and PCPs’ perspective.

#### Facilitators to Diabetes Web-Centric Information and Support Environment Usage

The PCPs appreciated that DWISE could be used as a teaching tool to teach diabetes-related self-management skills to their patients:

I try to guide my patients towards diabetes related self-management resources available...but really, there are silos of teaching that affects one’s ability to learn. DWISE is good...it is more comprehensive and is more relevant to a patient’s needs...this can help deal with these problems...I believe this should be accessible to most patients.

The participants felt that although there are many diabetes educational resources, they want DWISE type apps that consider a patient’s personal preferences and psychosocial concerns, when designing self-management strategies. Both patients and PCPs felt that there is more need for information about the psychological issues for patients with diabetes:

There should be apps that talk more about things like...distress, depression and psychology...I mean diabetes is hard, sometimes we are distressed and we need information and we rely a lot on Internet...

Finally, patients who were more engaged in their self-management felt that DWISE is an ideal tool for them, and they believed that using DWISE will be easier for them. Participants overwhelmingly stated that they would like some kind of technical support, such as online or face-to-face sessions on DWISE, to teach them how to use DWISE. They suggested that there should be a helpdesk or other resources to help them troubleshoot to facilitate use of DWISE.

#### Barriers to Diabetes Web-Centric Information and Support Environment Usage

From the PCPs’ perspective, time constraints were deemed to be a key barrier in the use of DWISE. PCPs felt that inclusion of DWISE-based intervention might not be feasible during the patient encounter because of the limited time that they have with their patients:

I love it...but can I do a good job with it? How can I incorporate this within the time restrictions?...at times it may not be conducive to my schedule...a patient gets just 15 minutes with the provider.

PCPs were also worried about liability related issues:

Ok, suppose I am using this app with my patients, what if I missed something or I fail to do what is expected of me? Would I be liable...what will be the impact?

PCPs also stated that including DWISE in their practice might result in additional work for them. Patients were worried that if they failed to achieve the goals that they have set through DWISE, they might lose respect in the eyes of the PCP, or disappoint them, and might feel burdened or stressed:

I mean respect is a two-way street...what if I don’t meet that goal...what would my doctor think about me?

Patients also stressed on preference for direct patient-provider contact:

Sometimes I just want to talk during an appointment with my doctors...maybe I don’t want to talk through an app during this time.

**Table 1:** Axial codes in each category. DWISE: Diabetes Web-Centric Information and Support Environment; PCP: primary care practitioner.

A priori category	Axial codes (number of open codes contained in each axial category)
Facilitators to DWISE usage	Technical support to facilitate DWISE usage (10)
	Teaching tool for patients (5)
	Need for personalized diabetes self-management apps (4)
	Need for information about psychological issues (2)
	Compliance with DWISE easier for engaged patients (1)
Barriers to DWISE usage	Practicality of DWISE due to PCP time constraints (4)
	Impact on patients who fail to achieve DWISE set goals (4)
	Age-related suitability (4)
	Practicality of DWISE because of technically challenged users (3)
	Preference for direct patient-PCP contact (3)
	Additional work (1)
	Liability-related issues (1)
Patient self-management	Potential to improve self-management and monitoring (3)
	Potential to modify behavior (1)
Patient education	Teaching tool for patients (5)
	Potential to improve patient awareness of disease (3)
Patient autonomy and preference	Patient autonomy in choosing self-management support delivery method (4)
	Power dynamic between patient and provider (2)
	Potential to improve patient empowerment (3)
Patient-provider communication	Insight into patient’s self-management practices (2)
	Potential to improve patient-provider communication (3)
	Preference for direct patient-provider contact (3)
Encounter-related issues	Impact on patient provider encounter (3)
	Practicality of DWISE due to PCP time constraints (4)
Professional roles and responsibilities	Professional roles and responsibilities around DWISE usage (2)
	Additional work (1)
Usability- or acceptability-related issues	Reminders to improve usability (1)
	System feedback (2)
	Information presentation in DWISE (2)
	Integration with other devices (2)
	Need for personal features in DWISE (3)

Both PCPs and patients felt that technology ineptness might be a deterrent to their use of DWISE:

One of my colleague is not tech savvy...there might be other providers like her. How can these people benefit from DWISE?...would they be interested?

PCP

One of the patients said:

I am not technologically adept, these are new and exciting...I like help with managing my diabetes...but there might be big learning curve for me.

Patient

#### Patient Self-Management

Participants felt that DWISE has the potential to improve diabetes self-management, especially given that mobile phones are ubiquitous and self-management plans formulated through DWISE can easily be integrated into the patients’ lives. Participants indicated that DWISE has the potential to improve diabetes-related monitoring:

Phone is ubiquitous, so more opportunities. I love apps for recording and monitoring...this can help me monitor my sugar.

Patient

One PCP remarked:

It helps me gain more information about diabetes-related behavior change and about my patient and both my patient and I can see if my patient is on the right track...we will have something to talk about next time we meet.

PCP

#### Patient Education

Participants felt that DWISE may help improve patient’s awareness of the disease and can be used as a teaching tool for patients:

DWISE makes me more aware...more informed...I feel like I want to know more so that I can better take care of myself.

#### Patient Autonomy and Preference

Patients felt that they should have autonomy in choosing self-management support delivery method. One patient said:

I don't believe that one size fits all...it is good to have platforms like apps...DWISE is easily available...it should not be made mandatory for every patient...I mean it has to be my choice.

A patient also stated that DWISE has potential to improve power dynamics between the patient and PCP and help patients gain more control over their diabetes management:

I feel balance of power is always in favour of my doctor...it’s not bad...but I like to be more involved...make decisions that fits my life...DWISE can give me more control.

In general, participants felt that using a tool such as DWISE might make them feel more empowered to self-manage their condition.

#### Patient-Provider Communication

Although appreciating that DWISE has the potential to provide better insights into their patients’ self-management practices, PCPs felt that DWISE could also improve communication between patients and PCPs around diabetes-related self-management. One of the PCPs said:

When a patient is first diagnosed with diabetes...DWISE can be a good avenue for discussion...about how a patient is feeling, what is it they want...how can they fit the self-management in their lives.

PCP

Patients also felt that DWISE could potentially help them to communicate personal issues that might affect their self-management practices and that otherwise would not come up during an appointment:

Doctors don’t live with diabetes...I live with diabetes...I have lived with diabetes for so long...this type of technology and apps can support me to better communicate with my doctor...what I am going through...why I am not able to follow proper diet or...not exercising...

#### Encounter-Related Issues

Although some PCPs expressed that it might not be practical to use DWISE during the encounter because of PCPs’ time constraints, other PCPs and patients expressed that DWISE can have a positive impact on the patient-PCP encounter in terms of shared decision making around the setting of SMART goals. PCPs underscored that a patient might be more prepared during the encounter:

Every patient is different...and self-management requirements vary so much...so patients coming prepared will be so good for the appointment...I think appointment time will be better spent.

Patients expressed that they will be more motivated to comply with plan set through DWISE to have a meaningful encounter:

There are higher problems that are not in my control...that might mix the schedule...but I will still try to do this or change it to have a better appointment...I’ll go to the appointment with something...

#### Professional Roles and Responsibilities

PCPs were unsure about the professional roles and responsibilities pertaining to the usage of DWISE in a clinical setting. They wondered how doctors, CDEs, and nurses would coordinate and collaborate to ensure that a tool such as DWISE can be used effectively:

How would this work...I mean how do we collaborate...should this be administered through a doctor or a nurse educator...who would monitor.

#### Usability- or Acceptability-Related Issues

Finally, participants offered some feedback regarding issues related to the usability and acceptability of DWISE. Although some participants suggested that there should be more reminders to help them comply with self-management plans and upcoming activities, others suggested that a user should be able to disable the reminder when he or she feels like. Participants commented on the information presentation in DWISE and suggested that there should be a better layout, too much text should be avoided, and it should be replaced by user-friendly features such as pictures and figures. They suggested that a good feature would be to have a space for users to type their notes in free text. Participants also expressed their desire to have some personal features included in the app, such as provision to include their “profile picture,” “personal profile information,” and “personal diabetes story.” Participants further suggested that DWISE should be integrated with other data collection devices, such as Fitbit, smart watch, and so on.

## Discussion

### Strengths

Digital health technologies have been effectively used for health information collection, information utilization, and sharing solutions. In this study, we have demonstrated that digital health applications can effectively and efficiently incorporate evidence-based health care knowledge to provide evidence-informed decisions or recommendations to support both health care professionals and patients. This is an ongoing work and DWISE is a proof of concept. Nevertheless, this work has provided a unique digital health solution to translate complex health care knowledge, that is, guidelines, clinical workflows, behavior models, educational content, and long-term care plans, in terms of easy-to-use, evidence-informed, point-of-care decision aids for both PCPs and patients. From a clinical perspective, the contribution of this research is the translation of specialized behavior change knowledge to family physicians and diabetes educators, thus enabling them to offer behavior change interventions to a larger population of patients with diabetes—at present, only one-third of Canadians with diabetes receive diabetes educational programs [66]. From the patients’ perspective, the contribution is a self-management program that engages and empowers them to manage their condition in a home-based and primary care setting as opposed to relying on visits to specialist clinics. From a health professional’s perspective, this work is the first attempt to empower and engage PCPs to administer behavior change counseling, and it provides a comprehensive readiness assessment and educational framework to educate PCPs about how to perform standardized behavior change counseling. The provision of PCPs facing behavior change support tools is a novel initiative and can potentially improve patients’ access to professional behavior change counseling.

A unique aspect of this research is the integration of paper-based medical knowledge, behavior change models, health care knowledge management methods, and mobile technologies to develop “intelligent and adaptive” mobile patient-centered solutions that are customizable to specific care contexts, users’ knowledge, and interests. The project has contributed a generic digital health strategy and technology, based on theoretical models that can be applied to a range of medical conditions, to deliver intelligent and ubiquitous health educational and decision aids. In the long term, we plan to extend the research to other chronic diseases where we will account for different disease-specific factors pertaining to the personalization of behavior change strategies. In the medium term, we would augment the research scope to incorporate other related metabolic conditions that are characterized by hyperglycemia such as prediabetes.

### Limitations

The 3 qualitative studies included small sample sizes. However, for usability studies, a small number of participants are deemed sufficient for determining the major usability issues [61,62]. Through our focus group study, our aim was to better understand the underlying barriers and facilitators to the use of DWISE for diabetes self-management in a shared decision-making setting, its impact on patient encounter and experiences, and its role in patient-provider communication. We do not know to what extent the sample we tested is representative; it is likely that we had a sample that is more favorable toward a digital health solution, as the participants volunteered to take part in this study. We sought the perspectives of a small number of participants after a 15-min demonstration of the system—we realize that maybe participants might have needed more time to adjust to DWISE because it was a new technology for them. As such, it is possible that participants might have missed or misinterpreted some of the features and functionalities offered by DWISE, which may have resulted in some of the responses being biased. Nevertheless, we draw confidence from the study methodology that ensured 2 researchers were present throughout the focus group study to provide clarifications and to alleviate any misconceptions participants may have had about the functionality of DWISE. We realize that this is a pilot study with a small sample size. Moreover, the PCP participants are mainly represented by the CDEs, which limits the generalizability of the study. Nevertheless, we are taking the feedback generated through these studies to improve the design, content, usability, and usefulness of DWISE framework. In the next stage, we plan to clinically evaluate DWISE for its effectiveness and safety in primary care settings, with the intent to disseminate it across the province of Nova Scotia.

### Conclusions

In this paper, we have presented a digital health solution to translate complex health care knowledge, that is, guidelines, clinical workflows, behavior change models, educational content, and long-term care plans, in terms of easy-to-use, evidence-informed, point-of-care decision aids for both PCPs and patients. The knowledge modeling methods and decision support technologies being developed are both scalable and generic in nature, such that they can be readily applied to computerize CPG for other chronic diseases to develop low-cost decision support aids that can standardize the care of chronic diseases and comorbidities at the primary care level.

DWISE has been evaluated for usability, functionality, usefulness, and acceptance in a shared decision-making environment through a series of qualitative studies. In general, for the PCP decision support tool, most usability problems were associated with the navigation of the tool and the presentation, formatting, understandability, and suitability of the content in the tool. For the patient support tool, most usability issues that were raised were related to the tool’s screen layout and design features, understandability of the content, clarity of the labels used, and navigation across the tool. With regard to the usefulness of DWISE in a shared decision-making environment, the most significant barrier from the PCPs’ perspective is the limited time PCPs have during an encounter and, and from patients’ perspective, the concern is the fear of failure to accomplish their goals to achieve diabetes control through behavior change interventions. In terms of facilitators, PCPs identified the potential of DWISE as a teaching tool for their patients, and the patients appreciated that DWISE provides personalized information especially on psychological issues that could be very useful to them. In general, participants felt that provision of technical support, especially to the elderly users and those who are not proficient in technology will facilitate the use of DWISE. Patients preferred that DWISE should not be made mandatory and should not completely replace the direct interactions with the PCPs, rather should be regarded as an additional support mechanism. Patients felt that DWISE may help them gain more control over their diabetes management, whereas PCPs suggested that it could assist them to gain more insight into a patient’s self-management practices. PCPs seemed unsure about their respective roles and responsibilities around DWISE usage. The results of these studies were used to guide the modification of DWISE in terms of its functionalities, screen layout and navigation, and content.

In conclusion, we contend that digital health technology, such as DWISE, that integrates a patient’s (clinical and behavioral) profile with CPG-based best evidence and SCT-based behavior change theories, when used in a shared decision-making environment, has the potential to improve self-management and increase sense of collaboration and trust in the care process. Our finding suggests a dynamic interplay between patients, PCPs, as well as systemic and technology factors in terms of the operationalization of the DWISE framework for diabetes management. However, we also believe that the implementation of an integrated framework such as DWISE, in a shared decision-making clinical environment, requires additional time for the technology to mature, technical innovation, organizational support for technology uptake, and a clear definition of professional roles and responsibilities.

## Abbreviations

BCI: Behavior Change Institute
BCO: Behavior Change Ontology
CDA: Canadian Diabetes Association
CDE: certified diabetes educators
CPG: clinical practice guidelines
DMC: Diabetes Management Centers
DWISE: Diabetes Web-Centric Information and Support Environment
HbA_1c_: glycated hemoglobin
PCP: primary care practitioner
SCT: social cognition theory
OWL: Web Ontology Language
SMART: Specific, Measurable, Action-Oriented, Relevant, Timely
TAP: Think Aloud Protocol
